# High resolution MRI imaging at 9.4 Tesla of the osteochondral unit in a translational model of articular cartilage repair

**DOI:** 10.1186/s12891-015-0543-0

**Published:** 2015-04-16

**Authors:** Lars Goebel, Andreas Müller, Arno Bücker, Henning Madry

**Affiliations:** Center of Experimental Orthopaedics, Saarland University Medical Center, Kirrberger Straße, Building 37, Homburg/Saar, D-66421 Germany; Department of Orthopaedic Surgery, Saarland University Medical Center, Kirrberger Straße, Building 37, Homburg/Saar, D-66421 Germany; Cartilage Net of the Greater Region, University of the Greater Region, Homburg/Saar, D-66421 Germany; Department of Diagnostic and Interventional Radiology, Saarland University Medical Center, Kirrberger Straße, Building 57, Homburg/Saar, D-66421 Germany

**Keywords:** High-field MRI, Sheep, Cartilage defect, Marrow stimulation, SGE 3D

## Abstract

**Background:**

Non-destructive structural evaluation of the osteochondral unit is challenging. Here, the capability of high-field magnetic resonance imaging (μMRI) at 9.4 Tesla (T) was explored to examine osteochondral repair *ex vivo* in a preclinical large animal model. A specific aim of this study was to detect recently described alterations of the subchondral bone associated with cartilage repair.

**Methods:**

Osteochondral samples of medial femoral condyles from adult ewes containing full-thickness articular cartilage defects treated with marrow stimulation were obtained after 6 month *in vivo* and scanned in a 9.4 T μMRI. *Ex vivo* imaging of small osteochondral samples (typical volume: 1–2 cm^3^) at μMRI was optimised by variation of repetition time (TR), time echo (TE), flip angle (FA), spatial resolution and number of excitations (NEX) from standard MultiSliceMultiEcho (MSME) and three-dimensional (3D) spoiled GradientEcho (SGE) sequences.

**Results:**

A 3D SGE sequence with the parameters: TR = 10 ms, TE = 3 ms, FA = 10 °, voxel size = 120 × 120 × 120 μm^3^ and NEX = 10 resulted in the best fitting for sample size, image quality, scanning time and artifacts. An isovolumetric voxel shape allowed for multiplanar reconstructions. Within the osteochondral unit articular cartilage, cartilaginous repair tissue and bone marrow could clearly be distinguished from the subchondral bone plate and subarticular spongiosa. Specific alterations of the osteochondral unit associated with cartilage repair such as persistent drill holes, subchondral bone cysts, sclerosis of the subchondral bone plate and of the subarticular spongiosa and intralesional osteophytes were precisely detected.

**Conclusions:**

High resolution, non-destructive *ex vivo* analysis of the entire osteochondral unit in a preclinical large animal model that is sufficient for further analyses is possible using μMRI at 9.4 T. In particular, 9.4 T is capable of accurately depicting alterations of the subchondral bone that are associated with osteochondral repair.

## Background

Non-destructive structural evaluation of the osteochondral repair tissue in experimental cartilage repair is challenging [1-3]. Translational large animal models play an important role, with especially the ovine stifle joint sharing morphological characteristics of the human knee [4-7]. However, visualisation of the morphology of the repair tissue of the entire osteochondral unit, often comprising only few millimetres in diameter, is complex even in such large animal models [1-3,8]. Moreover, specific alterations of the subchondral bone associated with cartilage repair have been recently described [4]. These include, for example, the upward migration of the subchondral bone plate, defined as elevation of the osteochondral junction into the cartilaginous repair tissue [4]. Also, intralesional osteophytes are often appearing as focal, newly-formed bone located apical to the original cement line [4]. Finally, subchondral bone cysts are also associated with marrow stimulating techniques [4]. As the extent of these changes is often small in relation to the defect size, their visualisation remains a problem using conventional imaging techniques. In this regard, the development of novel non-invasive tools such as high-field magnetic resonance imaging (μMRI) has the potential to significantly broaden the armamentarium to precisely assess experimental osteochondral repair [9,10].

Magnetic resonance imaging (MRI) is a clinical routine for non-invasive *in vivo* diagnostics of cartilage pathologies [11-16]. MRI scanners, mostly at field strength between 1.5 and 3.0 Tesla (T), have also been used for the evaluation of osteochondral repair studies in animals [17]. Over time, technique and applications have been continuously sophisticated. Of note, the development of μMRI scanners at 9.4 T allows for a detailed assessment of experimental cartilage repair, especially when dedicated transmit/receive coils for small samples are employed [18-21]. An increase in field strength directly correlates with a better signal-to-noise ratio (SNR) and higher resolutions, a main pillar when morphological MRI analyses are performed. Also reduced scanning time may be advocated. While μMRI offers a vast range of possible applications [13], higher radiofrequency (RF) energy deposition is applied resulting in warming of the samples and maintaining the field homogeneity is demanding [21-26]. In contrast to conventional experimental methods for assessing osteochondral repair, MRI permits a non-destructive and direct evaluation of osteochondral specimen without the often time-consuming need for decalcification or other processing. Similarly important, a multiplanar assessment of the entire reconstructed specimen is possible.

The purpose of this study was to explore with μMRI at 9.4 T the morphological appearance of the normal osteochondral unit and defect repair in a preclinical large animal model. Specifically, standard MRI sequences were adapted and optimized for the *ex vivo* imaging of small osteochondral samples. A specific aim of this study was to detect recently described alterations of the subchondral bone [4,27-29] associated with cartilage repair.

## Methods

### Animal experiments

For optimisation of *ex vivo* imaging protocols for small osteochondral samples at 9.4 T, 38 medial condyles of the stifle joint of 19 female ewes aged between 2 and 4 were used. The samples were part of a study on experimental osteochondral repair in a translational large animal model [29]. All animal experiments were conducted in accordance with the German legislation on protection of animals and the NIH Guidelines for the Care and Use of Laboratory Animals [NIH Publication 85–23, Rev. 1985] and were approved by the local governmental animal care committee [Tierschutzausschuss der Universität des Saarlandes, Homburg, Germany].

**Figure 1:**
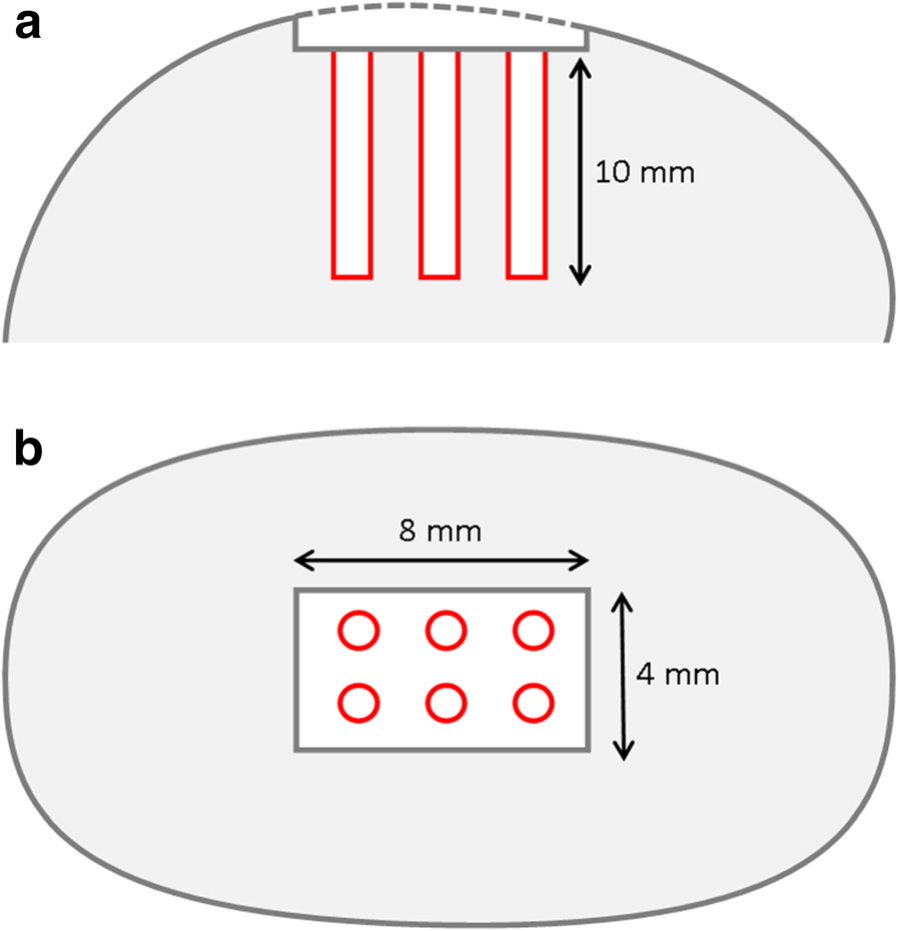
Schematic illustration of a medial fermoral condyle with a cartilage defect and drill holes. sagittal **(a)** and axial **(b)** cross section. In **(a)** the dashed line indicates the former level of articular cartilage. Each defect (4.0 × 8.0 mm) was treated with six drill holes (diameter 1.0 mm, length 10.0 mm).

Standardized, rectangular full-thickness chondral defects (size 4 mm width x 8 mm length) were created in the weight-bearing area of the medial femoral condyle in each stifle joint and treated with Pridie drilling by introducing six subchondral drill holes with a diameter of 1.0 mm into each defect using a Kirschner wire to a depth of 10 mm in a standardized manner (Figure 1) as described before [9,10,27,29]. Here, outmost caution was taken to meticulously remove the calcified cartilage from the subchondral bone [30,31].

Animals were allowed full weight-bearing immediately after surgery. Six month after surgery, the sheep were sacrificed in general anaesthesia and the osteochondral samples were subjected to gross examination. The 38 medial condyles were then explanted and the anterior two third of the condyles were put in 4% formalin for 48 h, then transferred to 70% ethanol and prepared for MRI investigation.

### Evaluation by 9.4 T μMRI

Explanted medial condyles were scanned in a 9.4 T μMRI developed for imaging of small animals (Biospec Avance III 9.4/20, Bruker Biospin, Ettlingen, Germany) with a gradient strength of 675 mT/m (BGA 12S gradient system) at room temperature. For imaging of osteochondral repair, an off the shelve circular polarized volume coil for imaging of the rat head or the mouse whole body with an inner diameter of 40 mm was employed. Maximum peak pulse power in send mode was 400 W. Maximum pulse duration at this power level was 5 ms. For maximum performance, the prescan protocol included wobbling of both channels and performing linear shims of 1st and 2nd orders. The samples were placed in the isocenter of the magnet with the defect oriented in orthogonal position to B0.

A rack and a versatile putty was used to assure a standardized and easy reproducible positioning of the samples while Parafilm M® (Pechiney Plastic Packaging Company, Chicago, Illinois, USA) served as cover to prevent samples from desiccation. Analysis in the ethanol solution used for sample preservation was not practicable as delineation to the adjacent articular cartilage layer was not possible.

Different materials were tested to serve as rack during the scanning progress. As a result, cuboid shaped blocs, sized 30 × 30 × 22 mm (length × width × height), were made out of oak wood, polyethylene, and polystyrene (Styrodur® C, BASF, Ludwigshafen, Germany). A bench drill (KTB 500, King Craft, Mülheim, Germany) was used to form a depression of 20 × 25 mm (depth × diameter) to gather the samples. Blu tack (UHU patafix®, Bühl, Deutschland), a versatile putty which can be removed again without residua, was used to fix the position of the specimen.

The osteochondral samples typically had a volume of 1–2 cm^3^ and were taken out of the ethanol solution for the scanning progress. To minimize acquisition time and warming of the samples and the employed coil system, readout direction was placed in alignment with the longest dimension of the scanned objects.

The coil system containing the samples was placed in the isocenter of the magnet and positioning was performed using a multislice gradient echo localizer sequence (repetition time (TR) = 100 ms, time echo (TE) = 6 ms, flip angle (FA) = 30^o^, number of excitations (NEX) = 1, slice thickness = 2 mm, interslice distance = 5 mm, bandwidth (BW) = 50000 kHz, field of view (FOV) = 8 cm × 8 cm, matrix size (MTX) = 128 × 128), generating sets of five subsequent images in three orthogonal planes. Based on this image stack, for each sample, a volume of interest was defined.

To identify the ideal measuring sequence, preliminary testing was performed with MultisliceMultiEcho (MSME) and spoiled GradientEcho (SGE) 3D imaging protocols and variation of parameters as e.g. TR or TE (Table 1).

**Table 1:** **Overview of different evaluated measuring parameters for the applied sequences**

**Sequence**	**TR**	**TE**	**FA**	**Voxel size tested (μm)**			**NEX**
MSME	10,000 ms	8.7 ms	180°	200 x 200 x 200	150 x 150 x 150	120 x 120 x 120	3 / 6 / 10 / 13
	10,000 ms	10.0 ms	180°				
	10,000 ms	12.5 ms	180°				
	10,000 ms	15.0 ms	180°				
	5,000 ms	8.55 ms	180°				
	5,000 ms	11.75 ms	180°				
	3,000 ms	11.75 ms	180°				
**SGE**	15.0 ms	3.5 ms	30°	200 x 200 x 200	150 x 150 x 150	**120 x 120 x 120**	3 / 6 / **10** / 13
	15.0 ms	3.5 ms	20°				
	15.0 ms	2.8 ms	20°				
	10.0 ms	3.5 ms	20°				
	10.0 ms	3.5 ms	10°				
	10.0 ms	3.0 ms	20°				
	**10.0 ms**	**3.0 ms**	**10°**				
	10.0 ms	2.5 ms	20°				
	10.0 ms	2.5 ms	10°				
	7.5 ms	3.5 ms	20°				
	7.5 ms	3.0 ms	20°				
	7.5 ms	3.0 ms	10°				
	7.5 ms	2.5 ms	20°				
	7.5 ms	2.0 ms	20°				

To evaluate the measuring parameters of the MSME and SGE sequences, the three different voxel sizes were always tested. TR = repetition time; TE = time echo; FA = flip angle; NEX = number of excitations. Voxel size and NEX were altered depending on image quality of the MRI scan, always starting with voxel size of 200 μm edge length and NEX = 10. Parameters in bold show the best fitting image sequence.

A special emphasis was laid to create voxels with identical edge lengths to allow for multiplanar reconstructions (MPR) in any desired plane without losing stereoscopic information. In a consecutive step the parameters for voxel size and NEX were altered to determine the best compromise between SNR, image quality and resolution in an affordable scanning time. Matrix size was adjusted each time to completely cover the samples.

After identification of best fitting parameters, all samples were scanned with this protocol. Consecutively, reconstructions in three orthogonal planes were performed in identical spatial resolution using the 3D software package supplied with the scanner (Paravision 5.1, JIVE tool, Bruker Biospin) and the resulting images were exported in the DICOM format and analyzed with ImageJ (version 1.45) [32]. Based on the acquisitioned images the samples were analysed for the morphological appearance of the native osteochondral unit and interesting associated features. The image analyses were performed by the senior author, a senior consultant orthopedic surgeon and the first author, a registrar for orthopedic surgery. Images were acquired by a biologist with special expertise in high-field MRI, following an established algorithm for image analyses. The orthogonal reconstructions were compiled by the first author. Measured variables include measuring time, diameter of drill holes and cysts, and intralesional osteophytes. Where appropriate, values are given as mean value ± standard deviation; and range. For the assessment of image quality and artifacts, the score developed by Hermann *et al.* was applied, assigning values from 1 point for a very poor quality, to 4 points for an excellent image quality [33].

## Results

### Supporting rack

Among all materials tested, the polystyrene rack, the Blu-Tack and Parafilm M® emerged to be MRI neutral without causing imaging artifacts and are therefore suitable materials for μMRI evaluation of osteochondral samples.

### Sequence optimization

Based on the evaluated measuring protocols for the applied sequences (Table 1), a 3D SGE sequence resulted in the best fitting for tissue contrast, image quality and resolution in a reasonable scanning time (Table 2).

**Table 2:** **Optimized imaging parameter for** ***ex vivo*** **analysis of osteochondral repair**

**Parameter**	**Optimal condition**
Slice orientation	Coronal
Read out gradient	Head-feet-direction
Phase encoding gradient	Left-right-direction
Excitation pulse shape	Sinc 10 H
Bandwidth	22,000 Hz
Pulse duration	1.0 ms
Read out effective spectral bandwidth	98,642 Hz
Read spoiler duration	1.03 ms; strength 30%.
Slice spoiler duration	1.0 ms; strength 40%.
Number of dummy scans	20
TR	10 ms
TE	3 ms
FA	10°
NEX	10
BW	98684.2 kHz

All consecutive scans of the osteochondral samples were performed with identical isotropic spatial resolution, resulting in an edge length of the voxels of 120 μm in three orthogonal planes. A matrix typically consisted of a set of 256 × 128 × 128 voxels.

### Measuring time

Preparation and positioning time of one single sample in a rack was about 30 min. Measuring time for one single sample applying a MSME sequence was between 5–7 h. Acquisition time of a SGE sequence was of 35–55 min.

### Imaging artifacts

**Figure 2:**
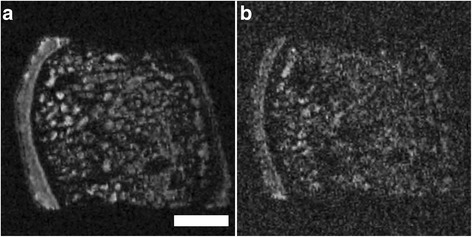
Overview of image quality obtained using μMRI at 9.4 T. Example of good **(a)** and poor **(b)** noise-to-signal ratio (NSR) and consequentially limited informative value. Axial reconstruction of same sample and identical plane. The SNR detoriated continuously when several consecutive scans were performed. SGE sequence, isotropic voxel size 120 μm^3^. Scale bar = 4 mm.

**Figure 3:**
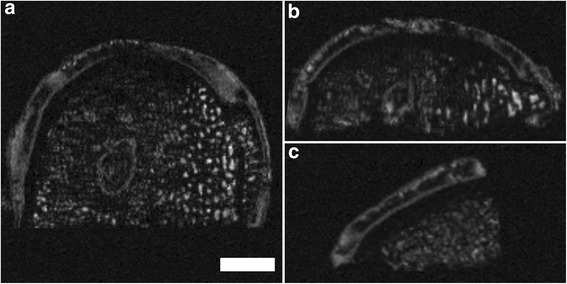
Example of poor image quality obtained using μMRI at 9.4 T. The images are blurred with interfolding and doubling of the surface. Axial **(a)**; coronal **(b)** and sagittal plane **(c)**; SGE sequence, isotropic voxel size 120 μm^3^. Scale bar = 4 mm.

Different imaging artifacts were observed during the scanning progress. Performing several consecutive scans resulted in a continuous warming of the MRI system, the coil and the sample (sample warming of approximately 2 to 3°C). This negatively influenced the quality of the acquired data sets, resulting in decreased SNRs (Figure 2). In this context, the MRI scans also were blurred, often showing a false interfolding and doubling of the surface (Figure 3). These phenomena vanished after cooling of the MRI system, necessitating several MRI scans of the same specimen in some cases.

### Subjective image quality

Overall, the subjective image quality of all scans was rated on a scale of 1 (insufficient) to 4 points (excellent) to be as 3.8 ± 0.5 (2 – 4).

### MRI morphology of the normal osteochondral unit at 9.4 T

**Figure 4:**
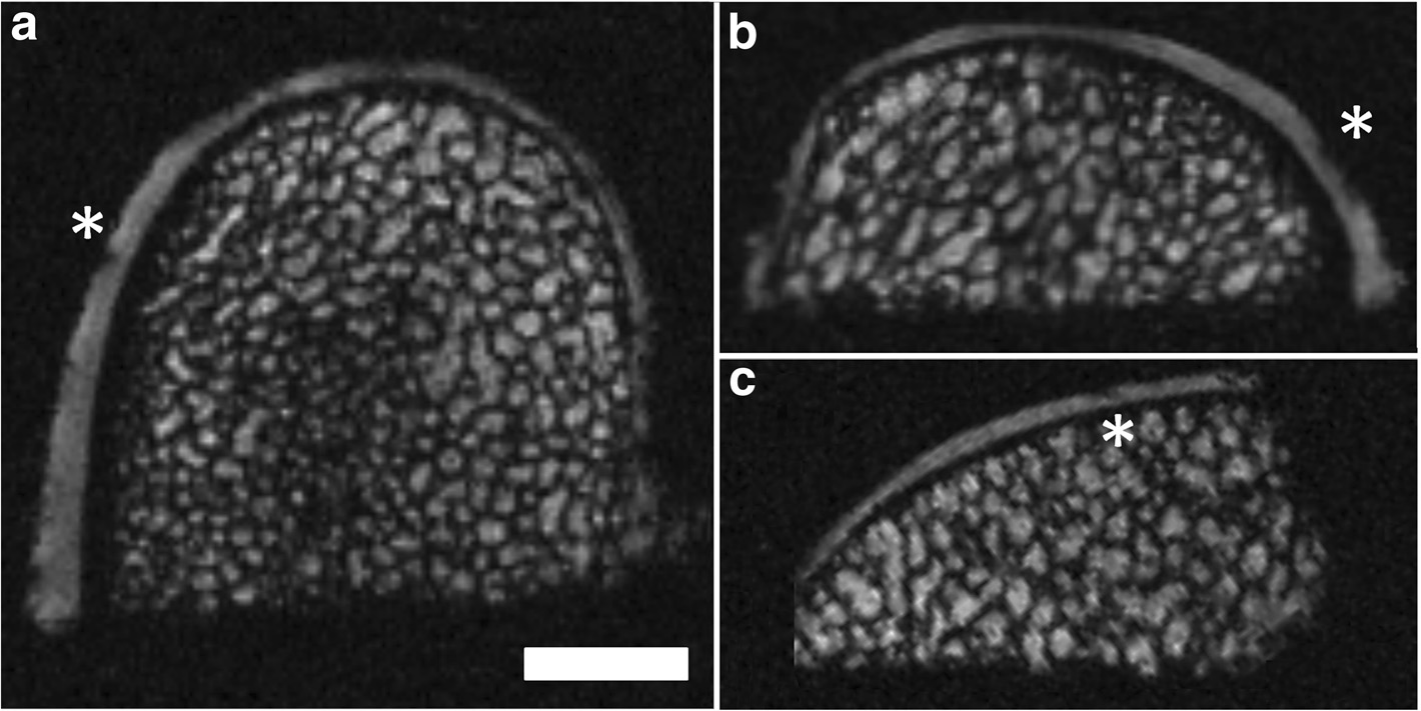
Example of a normal osteochondral unit obtained using μMRI at 9.4 T. Normal osteochondral specimen of the medial femoral condyle from an ovine stifle joint. Reconstruction in three orthogonal planes: axial **(a)**; coronal **(b)** and sagittal plane **(c)**. SGE sequence, isotropic voxel with 120 μm x 120 μm x 120 μm allowing multiplanar reconstructions without losing three-dimensional information. Note the minute artifacts (*) in the articular cartilage layer, caused by sample processing, and not resulting from defects of the osteochondral unit. Scale bar = 4 mm.

A clear depiction of the entire osteochondral unit was achieved using 9.4 T μMRI. In detail, the articular cartilage layer, the subchondral bone plate and the subarticular spongiosa with the fatty bone marrow were well distinguishable. The subchondral bone plate and subarticular spongiosa emerged as darkish, unintense structures between tissues with different shades of intensity. The signal intensity of the hyaline cartilage was hypointense, compared to the fatty bone marrow (Figure 4).

### MRI morphology of osteochondral repair after marrow stimulation at 9.4 T

**Figure 5:**
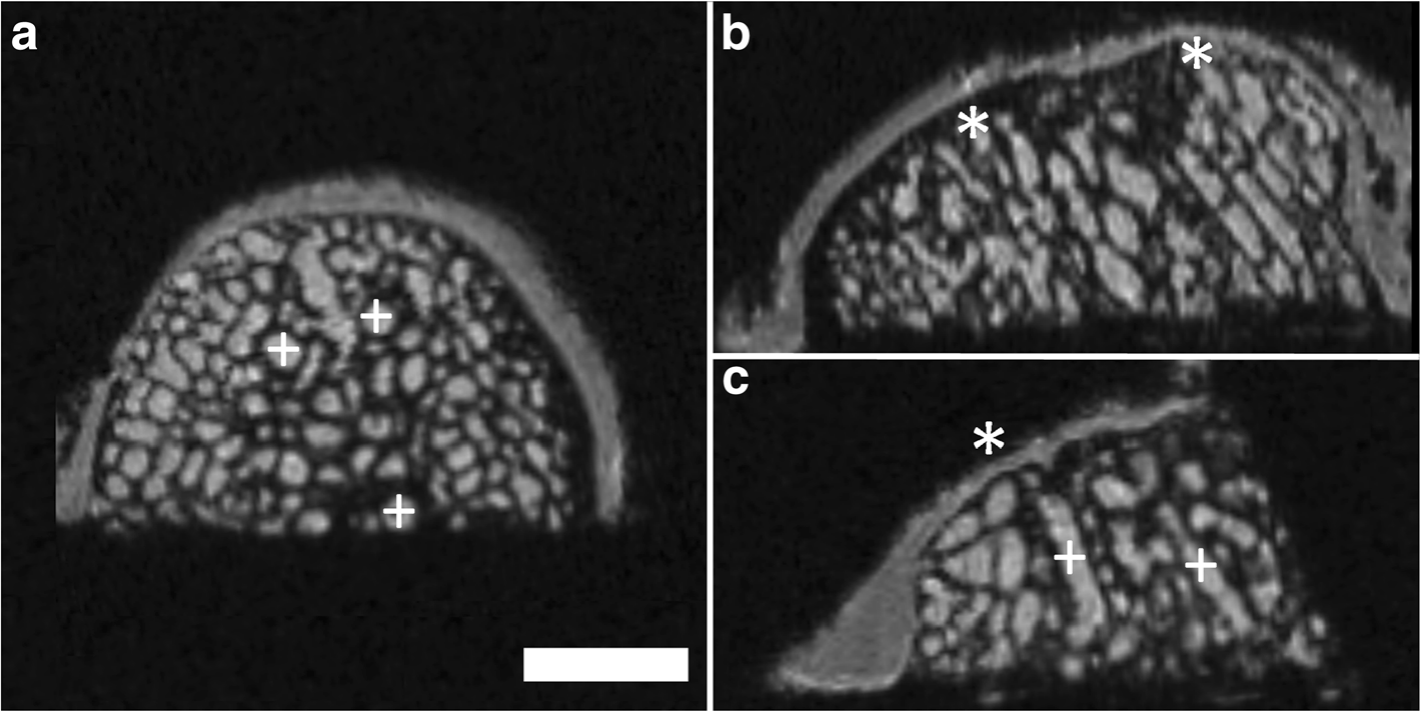
Example of good osteochondral repair 6 month after surgery obtained using μMRI at 9.4 T. Axial **(a)**; coronal **(b)** and sagittal plane **(c)**; SGE sequence, isotropic voxel size 120 μm^3^. Integration zone of the cartilage defect (*) and drill holes (+) are indicated. Note the relatively good filling of the defect and both horizontal and lateral integration of the cartilaginous repair tissue. Scale bar = 4 mm.

**Figure 6:**
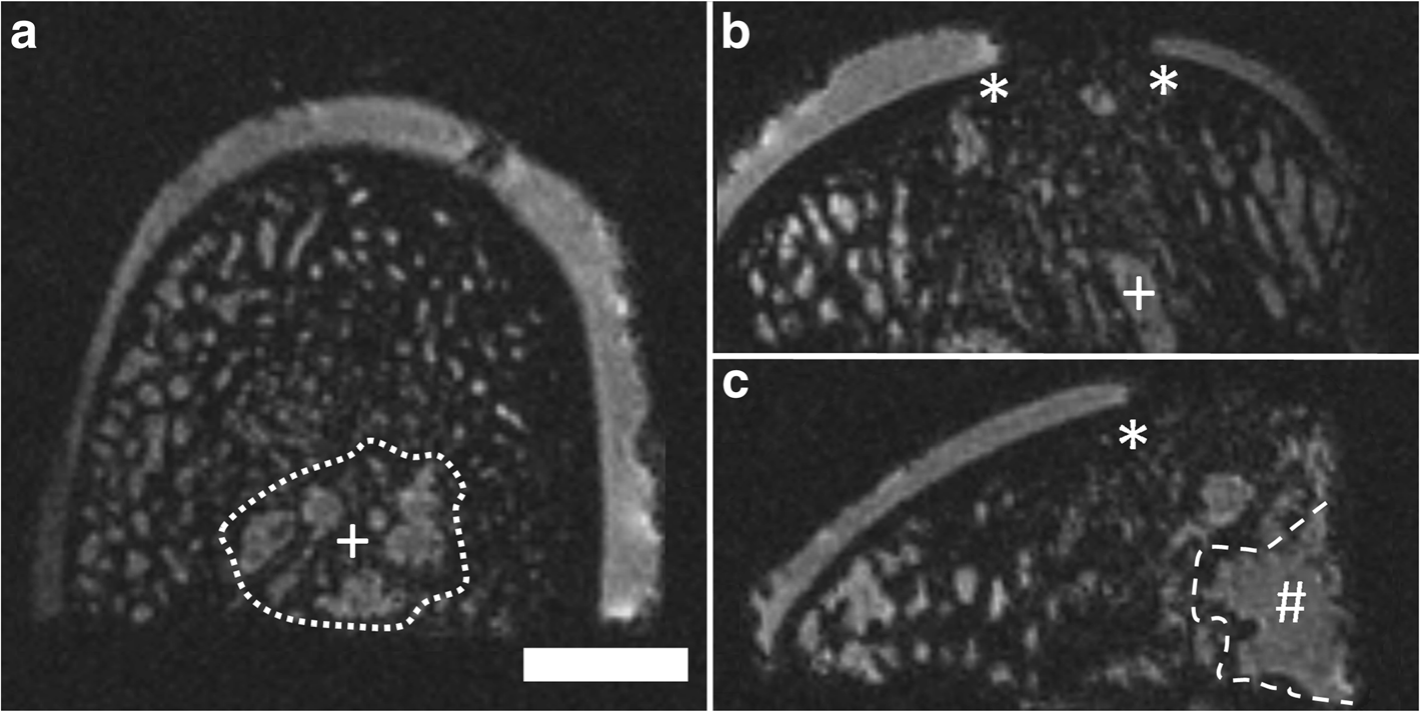
Example of failed osteochondral repair 6 month after surgery obtained using μMRI at 9.4 T. Cartilage defect (*) without repair tissue above level of the osteochondral bone plate; drill holes are filled with granulation tissue (+), a large cyst is partly visible (#). Axial **(a)**; coronal **(b)** and sagittal plane **(c)**; SGE sequence, isotropic voxel size 120 μm^3^. Scale bar = 4 mm.

Following marrow stimulation, the region of the articular cartilage defect was clearly distinguishable. Different grades of articular cartilage repair appeared very heterogeneously in μMRI. For example, osteochondral repair was rarely complete (n = 3; Figure 5); while sometimes no repair tissue with a defect filling above the level of the subchondral bone plate was apparent (n = 6; Figure 6). Often, insufficient defect fill between 0 and 25% of the repair tissue was seen (n = 17), while comparably less defects contained a repair tissue that filled between 25 and 75% of the defect (n = 12). Interestingly, the signal intensity of the repair tissue was not different from that of the hyaline cartilage in the applied imaging protocol.

### Persistence of subchondral drill holes

**Figure 7:**
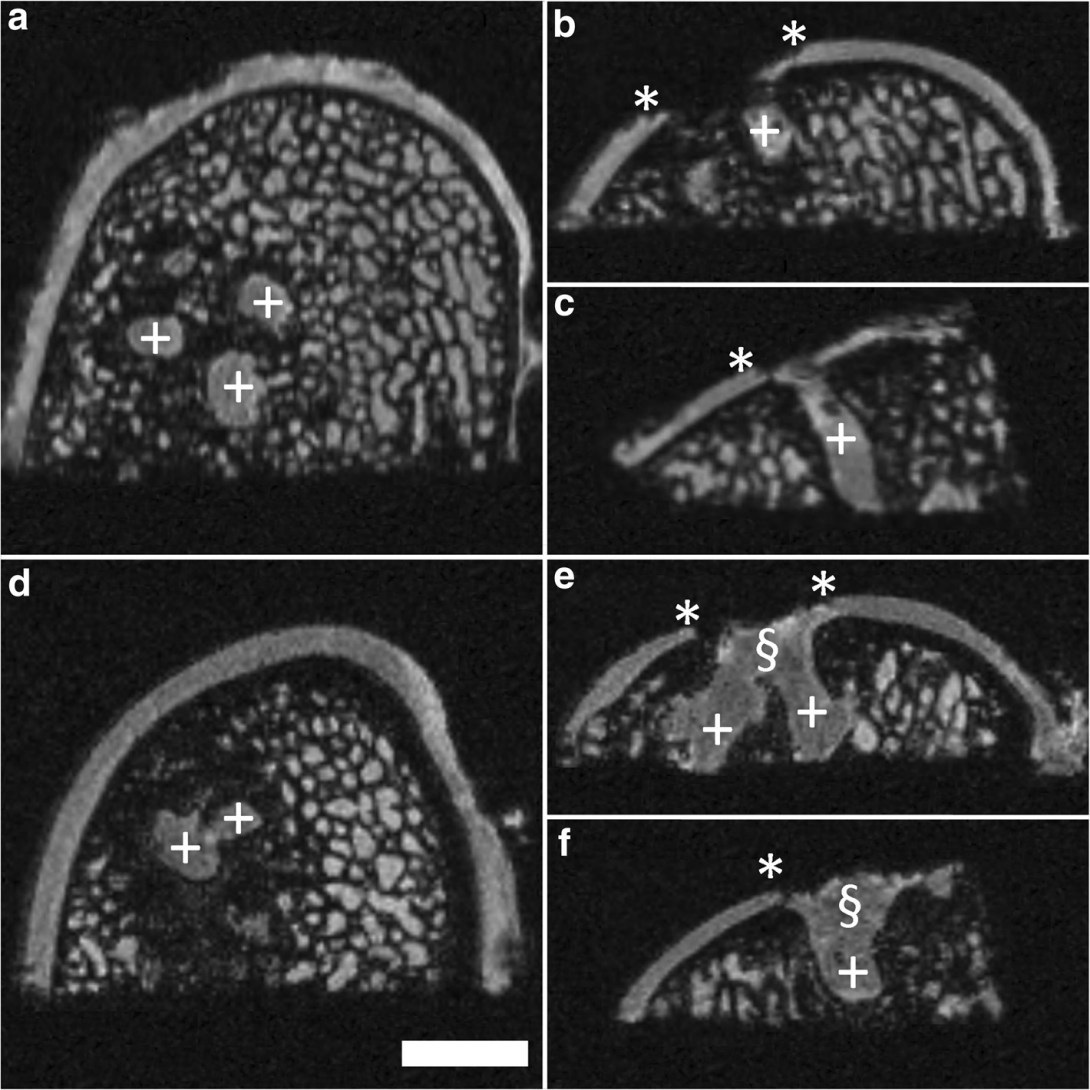
Persistence of drill holes at 6 month after surgery using μMRI at 9.4 T. Drill holes (+) were still identifiable 6 month after marrow stimulation. Most of them were filled with a repair tissue isointense to the cartilage repair tissue; some showing an infundibular morphology (§) when the defect extended to the subchondral bone. The repair tissue is only partially integrated with the adjacent articular cartilage. Asterisks (*) indicate extend of the cartilage defect. Axial **(a,d)**; coronal **(b,e)** and sagittal planes **(c,f)**; SGE sequence, isotropic voxel size 120 μm^3^. Scale bar = 4 mm.

At the time of surgery in each defect (n = 38) 6 subchondral drill holes (diameter: 1.0 mm) were introduced. After 6 month *in vivo*, subchondral drill holes were still identifiable in all specimens. They emerged as cylindrical structures within the subarticular spongiosa with a minimum length of 5.0 mm and a diameter between 0.9 and 2.0 mm. In all observed cases, a tissue which was isointense to the repair tissue filled the subchondral drill holes (Figure 7).

### Subchondral bone cysts

**Figure 8:**
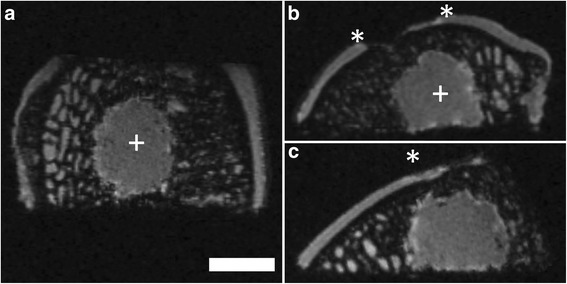
Example of subchondral bone cysts at 6 month after surgery obtained using μMRI at 9.4 T. Subchondral bone cysts (+) that are located in the subarticular spongiosa were commonly observed after marrow stimulation. Asterisks (*) indicate extend of the cartilage defect. Axial **(a)**; coronal **(b)** and sagittal plane **(c)**; SGE sequence, isotropic voxel size 120 μm^3^. Scale bar = 4 mm.

**Figure 9:**
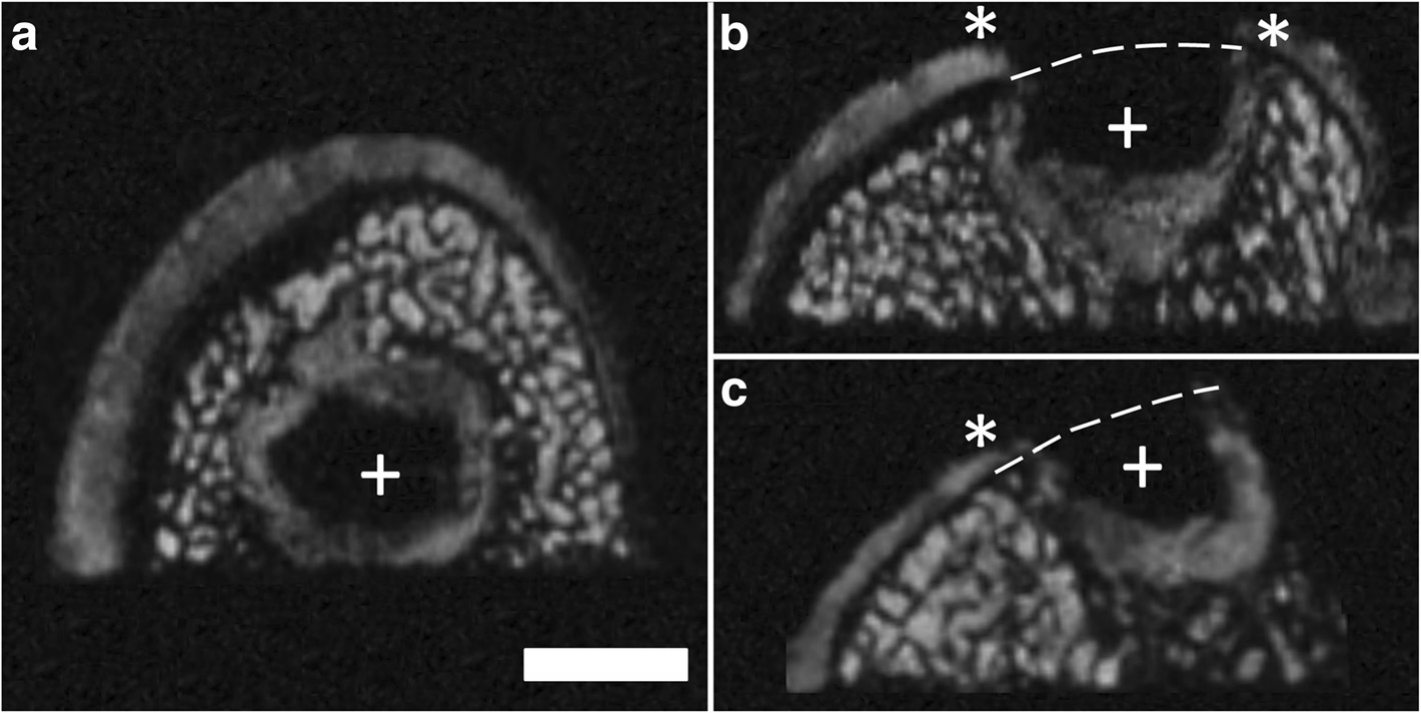
Formation of large osteochondral defect at 6 month after surgery using μMRI at 9.4 T. A large osteochondral defect (+) has formed six month after failed marrow stimulation of full-thickness chondral defect. The development of this large subchondral cyst in the subarticular spongiosa was associated with a subsequent collapse and complete resorption of the subchondral bone plate at the basis of the cartilage defect (dashed line). Asterisks (*) indicate the extent of the cartilage defect. Axial **(a)**; coronal **(b)** and sagittal plane **(c)**; SGE sequence, isotropic voxel size 120 μm^3^. Scale bar = 4 mm.

Subchondral bone cysts were defined as structures within the subarticular spongiosa with a diameter > 2.0 mm (n = 17; 4.2 ± 1.3 mm, 2.3 - 6.1 mm; Figure 8). They were either covered with a lamina surrounding a void or completely filled with a tissue isointense to the repair tissue. Some of the cysts completely undermined the treated cartilage defect while in others the entire subchondral bone plate collapsed (Figure 9).

### Sclerosis of the subchondral bone plate and subarticular spongiosa

**Figure 10:**
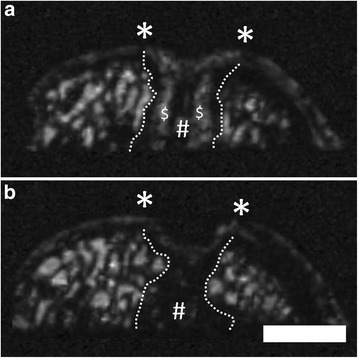
Sclerosis of the subarticular spongiosa at 6 month after surgery using μMRI at 9.4 T. Sclerosis of the subarticular spongiosa (# - area between dashed lines) was observed in 95% of the osteochondral samples after marrow stimulation. Asterisks (*) indicate the extent of the cartilage defect. Persisting drill holes ($). Subsequent coronal planes **(a, b)**. SGE sequence, isotropic voxel size 120 μm3. Scale bar = 4 mm.

A sclerosis of the subchondral bone plate and subarticular spongiosa was detected in the majority of the osteochondral specimen (n = 36; Figure 10). It was described as thickening of the subchondral bone plate or as predominance of the subarticular spongiosa, supplanting the fatty bone marrow. Rarely, the subchondral bone was completely restored following marrow stimulation (n = 1).

### Advancement of the subchondral bone plate

An advancement of the subchondral bone plate with resulting upward-migration of the osteochondral junction above the normal level was never observed at 6 month after marrow stimulation.

### Intralesional osteophytes

**Figure 11:**
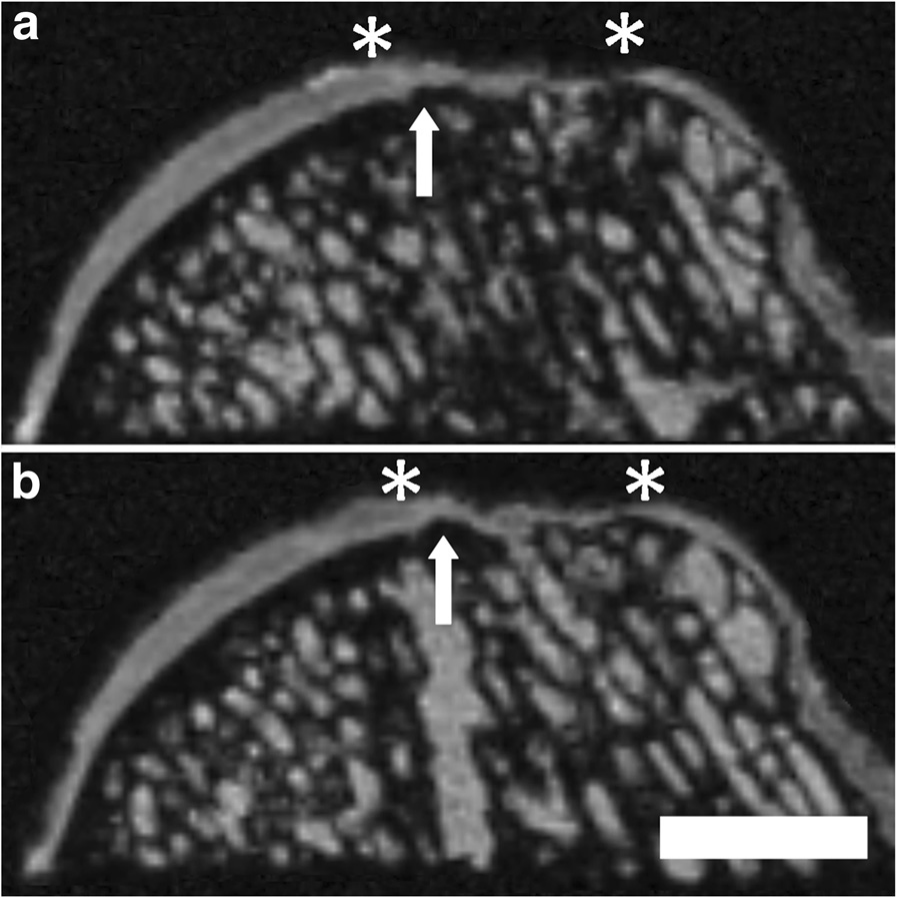
Formation of intralesional osteophytes at 6 month after surgery using μMRI at 9.4 T. Intralesional osteophytes (arrow) that arise from the subchondral bone plate into the cartilaginous repair tissue were observed in four osteochondral samples after marrow stimulation. Asterisks (*) indicate the extent of the cartilage defect. Subsequent coronal planes **(a, b)**. SGE sequence, isotropic voxel size 120 μm3. Scale bar = 4 mm.

Intralesional osteophytes were sometimes present (n = 4). They always occurred in the integration area, the intersection between the cartilage defect area and the native osteochondral unit. The mean height of the intralesional osteophytes was 0.4 ± 0.2 mm (0.2 - 0.4 mm; Figure 11). Intralesional osteophytes were never observed in the centre of the defects.

## Discussion

The aim of this study was to explore the advantages of 9.4 T μMRI as a new experimental tool to assess the entire osteochondral unit in a translational model. An overview of the spectrum of image findings associated with osteochondral repair is provided, describing the advance of imaging compared to conventional MRI, and to shed light on potential problems and pitfalls. The data demonstrate that μMRI at 9.4 T allows for a rapid and non-destructive assessment of osteochondral repair. μMRI is capable of achieving spatial resolution of 120 × 120 × 120 μm and below. Of note, no preliminary sample processing is necessary. MPR of isotropic voxels enable to examine the complex structure of osteochondral samples and repair in any desired plane without losing stereoscopic information. Visualisation and discrimination of articular cartilage, repair tissue, subchondral bone plate and subarticular spongiosa within the osteochondral unit is possible. Alterations of the subchondral bone that are associated with cartilage repair can be detected. Thus, μMRI emerges as a highly useful tool to assess osteochondral repair.

MSME sequences gave better SNR, although scanning time was several times longer. This made MSME sequences impracticable for larger study populations as scanning time in μMRI is often limited. For this reason, a 3D SGE sequence was chosen as alternative to MSME. With this technique, higher numbers of imaging experiments could be combined for generation of high resolution datasets in a straightforward time frame. As well an ample FOV selection was facilitated, while a TR/TE combination tested for optimum tissue contrast could be retained for all of the samples. Although 3D techniques employ two phase encoding steps, phase wrapping could be avoided.

In the microcomputed tomography (μCT) analysis of the subchondral bone, as described by Orth *et al.* [29], in 12 out of 19 condyles (63%) subchondral bone cysts were observed while in five defects (26%) intralesional osteophytes were found. In contrast, by 9.4 T μMRI analysis, subchondral bone cysts were detected in 17 out of 38 (45%) condyles and intralesional osteophytes in five out of 38 (13%) condyles. The different rates between both imaging techniques may in part be caused by different spatial resolution. While voxel size for the μMRI assessment was 120 μm edge length, samples in the μCT were scanned with a resolution of 15 μm. Especially, the mainly small intralesional osteophytes are then easier to display. *Vice versa*, with μCT only osseous structures are visible depicting the real extent of a subchondral cyst while with μMRI analysis also the lining of the cyst is visible, making it difficult to determine the real extent of the cyst.

This study revealed two major MRI artifacts. First, loss of signal intensity in cartilage was observed next to small areas of cartilage defects in nearly all samples, caused by the different magnetic susceptibilities of adjacent air and the surrounding tissue. This effect was exacerbated by low signal intensities, but not prominent in homogenous areas with proper lining of the cartilage layer. Second, embedding the samples in an ethanol solution during scanning progress made it impossible to distinguish the cartilage surface from the fluid environment.

Occasionally, SGE sequences resulted in images with particularly low signal intensity, respectively SNRs that could not be compensated immediately by resetting the imager with the standard prescan procedures, or elaborated shimming procedures. This effect can in part be attributed to B1 inhomogeneity caused by warming of the circular polarized volume coil used for both transmitting and receiving. However, due to restrictions in imaging time, measurement of B1 fields before and after imaging experiments were not performed.

Also, loss of signal intensity was often evidenced when repetitive imaging had to be performed on a given sample. Therefore we assume, to a certain extent, sample degeneration after repetitive scans [34,35]. In the future, samples might be frozen or kept in sterile phosphate buffered saline solution until μMRI assessment is completed and preserved in formalin thereafter.

On the basis of the applied MRI scanner with a bore size of 200 mm and additionally applied coiling systems, only *ex vivo* evaluation of osteochondral samples of sheep were possible. Consecutively, parameters as perfusion or bone marrow oedema could not be assessed. A comparison to experiments with a standard 3.0 T clinical scanner was not performed. Upcoming studies will also have to focus on different cartilage repair techniques as well as on advanced MRI techniques. An increased sensitivity to susceptibility effects may be used for susceptibility weighted-imaging which may allow gaining completely new contrast possibilities at higher field-strength [17,36-38]. While clinical MRI scanners at 1.5 T or 3.0 T usually rely on protons, other nuclei as ^23^Na, ^31^P or ^17^O may allow a more specific diagnosis because of a closer relationship to the pathology [36,39]. Nuclei other then protons are less sensitive and provide lower signals, resulting either in larger voxel size or increased measuring time, or both. Here, MRI with higher field strength of 7.0 T or 9.4 T may play a key role to foster clinical applications of non-proton MRI [40-42]. When the field strength is enlarged, proton relaxation times of the tissues change, as e.g. observed for T1 [22,36,37]. However, in this *ex vivo* analysis the main focus was laid on the increased SNR allowing for a reduction in spatial resolution. Taken together, a spatial resolution with voxels of 120 μm edge length were created while a 3D SGE sequence (Table 2) was found to give the best ratio between image quality and scanning time. Acquisition of 3D data allowed for multiplanar reconstructions without losing steric information.

To date only very few studies at all investigated the capabilities of μMRI at 9.4 T to identify pathological alterations of the osteochondral unit [9,10,43]. Transverse relaxation time (T_2_) was correlated with the overall volume of repair tissue in a rabbit cartilage defect model [43]. In a large animal model, macroscopic [9] and histological experimental osteochondral repair [10] were correlated with the Magnetic Resonance Observation of Cartilage Repair Tissue (MOCART) score at 9.4 T. Here, the five tested macroscopic scores exhibited high intra- and interobserver reliability and high internal correlation. When the individual parameters of the different macroscopic scores were correlated, the parameters “defect fill” and “total points” reflected well the data from the two-dimensional (2D) MOCART score assessment. Also, key histological categories “defect fill” and “total points” of both an elementary and a complex histological scoring system for experimental osteochondral repair [44] could reliably be determined by 9.4 T μMRI using either the 2D or three-dimensional (3D) MOCART system, while the 3D MOCART score reliably assessed the category “subchondral bone plate” of the Sellers histological scoring system [10].

A possible limitation of this study was that *ex vivo* analyses were performed. Parameters as bone marrow edema, a relevant cause of prolonged pain in clinical routine, e.g. as observed after surgery, could not be performed [45-47]. However, the focus of this study was the morphological description of experimental osteochondral repair in a large animal model, and joints of the sheep were too large to be accommodated by the μMRI scanner with a bore size of 200 mm. Subchondral drilling is easier to standardize in an experimental setting [27] than microfracture, which is used more often than drilling in the clinical routine. Advantages of μMRI include a possible visualization of the osteochondral unit immediately after sacrifice. A morphological non-destructive examination of osteochondral samples without further processing is possible allowing to discriminate between articular cartilage, repair tissue, subchondral bone plate and subarticular spongiosa within the osteochondral unit. Nonetheless, histological evaluation remains the gold standard for the evaluation of experimental articular cartilage repair. Future studies using 9.4 T μMRI will shed more light on the natural history of untreated full-thickness defects and compare different cartilage repair procedures. Another focus will be to examine the repair of sub-acute and chronic lesions [48], as most patients with articular cartilage defects have a history of symptoms for at least several weeks prior to the initiation of reconstructive surgical treatments.

## Conclusion

High-field MRI at 9.4 T allows for a detailed non-destructive *ex vivo* analysis of the entire osteochondral unit in a preclinical large animal model sufficient for further analyses. In particular, 9.4 T is capable of depicting alterations of the subchondral bone associated with osteochondral repair. Therefore, μMRI offers new avenues to examine experimental osteochondral repair.

## Abbreviations

2D: Two-dimensional
3D: Three-dimensional
μCT: Microcomputed tomography
μMRI: High-field magnetic resonance imaging
BW: Bandwidth
FA: Flip angle
FOV: Field of view
MOCART: Magnetic Resonance Observation of Cartilage Repair Tissue
MPR: Multiplanar reconstructions
MRI: Magnetic resonance imaging
MSME: MultiSliceMultiEcho
MTX: Matrix size
NEX: Number of excitations
NSR: Noise-to-signal ratio
RF: Radiofrequency
SGE: Spoiled GradientEcho
SNR: Signal-to-noise ratio
T: Tesla
T_2_: Transverse relaxation time
TE: Time echo
TR: Repetition time
